# No Evidence for Curiosity‐Driven Information Selection Advantage in Infants’ Novel Word Learning

**DOI:** 10.1111/desc.70101

**Published:** 2025-11-28

**Authors:** Marina Bazhydai, Malcolm K. Y. Wong, Elena Constanze Altmann, Samuel David Jones, Gert Westermann

**Affiliations:** ^1^ Psychology Department Lancaster University Lancaster UK; ^2^ Georg‐August‐Universität Göttingen Germany; ^3^ Department of Psychology Bangor University UK

## Abstract

The cognitive mechanisms and benefits of active learning in early child development are poorly understood. The current study investigated 20–23‐month‐old infants’ curiosity‐driven information selection in a novel word learning task, designed to identify any potential advantage for active learning over passive learning. In a gaze‐contingent eye‐tracking paradigm, infants in one condition were given the opportunity to structure their own information seeking to actively create word learning opportunities for themselves, while infants in two other conditions engaged in learning novel words passively. Infants’ learning of word‐object associations was compared across active and passive learning paradigms. The results indicate no advantage of active information selection on retention of novel words above and beyond passive learning, with infants across all conditions retaining novel words above chance. This study provides a crucial insight advancing our understanding of early word learning, and of the mechanisms and benefits of active, curiosity‐based learning in infants.

**Summary:**

- We investigated the effect of active, curiosity‐driven word learning, as compared to passive word learning, on infants’ label recognition.
- Infants’ self‐motivated information selection was tested using a novel word learning task in a gaze‐contingent eye‐tracking paradigm.
- Self‐motivated information selection had no effect on early word learning above and beyond passive learning, with infants across all conditions retaining novel words above chance.
- This provides novel insights into infants’ active and passive learning for language acquisition.

## 1 Introduction

The way in which infants learn their first words, and the mechanisms underlying this ability, have received considerable attention in developmental research (Westermann and Mani 2017; Mani and Ackermann 2018). A typical way to study this early stage in language acquisition is by showing infants novel objects live or on a computer screen and repeatedly pairing them with novel labels. In a subsequent test phase, recognition of these word‐object mappings is then assessed by presenting two or more of the objects side‐by‐side and measuring infants’ looking or pointing at the object corresponding to a heard label. Such research has shown that infants are able to rapidly map a limited number of novel labels to novel objects from around 14 months of age (e.g., Schafer and Plunkett 1998; Werker et al. 1998; Mani and Plunkett 2008). Nevertheless, this type of research sees infants largely as passive recipients of information (in this case, word‐object correspondences), the order and presentation duration of which are decided a priori by the experimenter. However, in the real world, infants act as curious learners who actively explore their environment and seek out information that satisfies their curiosity at a specific point in time. The current study investigated a potential advantage of active as compared to passive word learning, by giving infants an opportunity to structure their own information seeking to actively create word learning opportunities.

In contrast to passive learning, the curiosity‐driven learning approach (defining curiosity as active, self‐motivated search for information for its own sake) focuses on infants’ constructive engagement with available information, such as selectively attending to available information that is best suited for learning at a given moment, and seeking new information to guide their own knowledge acquisition (for reviews, see Bazhydai, Twomey, et al. 2020; Begus and Southgate 2018; Kidd and Hayden 2015). Computational modelling (Twomey and Westermann 2018) has suggested that successful learning is driven by an interaction between available information, the learner's prior knowledge, and the learner's readiness to learn a specific piece of information, and that learning is therefore enhanced when the learner is actively involved in selecting to‐be‐learned information compared to when receiving information at the will of the teacher or experimenter. The learning progress theory of curiosity posits that active information‐sampling (or curiosity‐driven learning) evolved as a mechanism to systematically reduce uncertainty in the environment and maximize information gain, resulting in optimal learning (Oudeyer and Smith 2016). Initially formulated through the use of developmental robotics models (Gottlieb et al. 2013), recent support for this theory comes from computational models (Twomey and Westermann 2018) along with corroborating evidence from infants (Poli et al. 2020) and adults (Ten et al. 2021). Thus, active learners need not have metacognitive awareness of their self‐motivated learning and information‐sampling strategies to enable efficient learning.

Broadly, empirical evidence has supported the benefits of active learning in adults and older children, demonstrating that having control over informational input enhances attention and memory and leads to faster and more robust learning (for reviews, see Gureckis and Markant 2012; Markant et al. 2016). For example, retention of information is improved when adults and children are curious about this information (Fandakova and Gruber 2020; Kang et al. 2009) and when 8–11‐year‐old children are able to control the order and pacing of learning during a memory task (Ruggeri et al. 2019). It could be argued that such self‐directed information sampling may enable optimal learning by being strategic, efficient, and systematic rather than random (Kachergis et al. 2013; Meder et al. 2020; Pelz and Kidd 2020; Zettersten and Saffran 2021), leaving open the question whether there might be optimal learning sequences that generalize across individuals.

Studies have shown that infants are able to self‐direct their visual attention to upcoming stimuli to optimize their information seeking (e.g., Kidd et al. 2012; Poli et al. 2020), and that visual and manual exploration of objects creates optimal conditions for learning labels, functions and categories (Smith et al. 2018). Furthermore, infants actively use non‐verbal communicative cues such as pointing and social referencing to express interest in specific information. For example, in situations of referential uncertainty about novel objects, even infants in their first year seek disambiguating labeling information from knowledgeable people (Bazhydai, Westermann, et al. 2020; Begus and Southgate 2012; Boundy et al. 2019). Such active information seeking in social contexts leads to enhanced learning outcomes: when infants pointed to objects prior to a learning phase, they retained information about those objects (e.g., labels or functions) provided by their social partners better than when they were instead taught about the objects that they did not actively select (Begus et al. 2014; Lucca and Wilbourn 2018).

In the context of early language acquisition specifically, curiosity‐driven active learning has also been argued to play an important role (Tamis‐LeMonda et al. 2018; Twomey and Westermann 2019), such that young children's vocabulary development, instead of being solely shaped by the linguistic input and environment, is driven by interactions between which words they already know and which new words they are interested in learning (Borovsky et al. 2016; Mani and Ackermann 2018). For example, 30‐month‐old toddlers retained more word‐object associations when they were given an opportunity to learn novel labels from the natural category of their interest (e.g., cars or animals), as opposed to objects in which they were not interested (Ackermann, Hepach, et al. 2020). Word learning in 20‐month‐olds was shown to be particularly successful when parents named an object that was in the infant's full direct view (as evident from the use of head mounted cameras; Pereira et al. 2014), which can be interpreted as social optimization of infants’ self‐directed learning by providing information when the child is ready to actively and effectively engage with it. Similarly, preliminary evidence with 3–5‐year‐old children (Partridge et al. 2015) showed that novel label recognition was enhanced following a learning phase that allowed children to learn labels actively by selecting novel animated toys on a touch screen, as compared to passively observing the labeling events, without an opportunity to make such active selections. Furthermore, accumulating evidence suggests that self‐directed word learning is driven by preschoolers’ awareness of their vocabulary gaps and the ability to pose questions to others about the meanings of words (Jimenez et al. 2018; Ronfard et al. 2018). Collectively, the reviewed lines of research highlight the beneficial role of self‐directed information sampling for learning outcomes.

However, emerging developmental evidence in this line of research so far lacks consistency. Zettersten and Saffran (2021) report inconclusive results about the relationship between active sampling and word learning in children aged 3–8 years. Likewise, in a touch‐screen study with 2‐to‐3‐year‐olds, no advantage of an active over passive word learning condition was found (Ackermann, Lo, et al. 2020). It therefore remains unknown whether enabling infants to actively control their learning process leads to better learning of word‐object associations. On a broader scale, further research is needed to understand the effect of active and passive learning approaches. Despite the demonstrated benefits of pedagogically guided play and exploration in early childhood (Yu et al. 2018; Weisberg et al. 2016), and of active learning and independent discovery for older children in a variety of learning contexts (e.g., Dean and Kuhn 2007; Saylor and Ganea 2018), traditional pedagogical learning environments often ignore children's natural curiosity, instead highlighting the effects of direct didactic instruction (e.g., in science education; Klahr and Nigam 2004).

### 1.1 Study Motivation

The challenge in studying word learning in infancy from a curiosity‐based learning perspective is to develop settings that enable infants to make active choices about the information they want to obtain, while retaining enough control over the environment to precisely characterize the nature of the infants’ information selection and its effect on learning. Here we developed such an approach by utilizing a gaze‐contingent paradigm to investigate infants’ visual exploration patterns, as opposed to explicit behavioral cues to active information selection such as choices, points or questions. Thus, infants were given an opportunity to generate their own learning sequences and select what to learn about and when to learn it, without any constraints—a level of control that goes beyond mere selective attention allocation to start a learning sequence as opposed to passively observing the learning episode (as in Ackermann, Lo, et al. 2020; Partridge et al. 2015).

Our paradigm capitalized on infants’ gaze rather than manual actions as an expression of curiosity, building on research with adults’ saccadic eye movements as visual information sampling (Baranes et al. 2015). Even 2‐month‐olds have been shown to coordinate their sucking behavior when it is contingent on receiving preferable visual presentation (e.g., “suck‐for‐clear” rather than for blurry pictures; Kalnins and Bruner 1973), which can be interpreted as a motivation for information gain. A recent surge in studies using gaze‐contingent procedures has successfully evaluated infants’ attention, motivation and learning (Keemink et al. 2019; Kenward 2010; Miyazaki et al. 2014; Sučević et al. 2021; Tsuji et al. 2020; Wass et al. 2011; Zettersten 2020; Wang et al. 2012).

Importantly, our paradigm was not dependent on the learners’ metacognitive awareness of learning goals, their ability to monitor their own information gain progress (as in Partridge et al. 2015, where preschool children had to learn 15 labels in a self‐paced task before the training phase ended), or on the potential extrinsic motivation to succeed in the learning task (as in Markant et al. 2016, and Ruggeri et al. 2019, where children were explicitly asked to remember as many objects as possible), making it highly suitable for infants and allowing us to shed light on the earliest emerging word learning mechanisms. Overall, this research has a potential to not only advance our understanding of early word learning as one of the foundations of language acquisition, but also our understanding of the general mechanisms and benefits of active, curiosity‐based learning in infants.

### 1.2 The Current Study

The current study addressed whether enabling infants to actively control their learning process leads to better learning of word‐object associations. Specifically, we investigated whether early word learning is enhanced when infants could actively choose the novel object for which they wanted to hear the novel label, as well as when they wanted to hear this label. To address this question, we used gaze‐contingent eye tracking to enable infants to exert choice over which objects presented on a screen were labeled at a certain point in time. As we were interested in whether the freedom to select information benefits word learning, we contrasted this active learning (Curiosity) condition with two passive learning control conditions. In one control condition (Random control), which is akin to traditional ways of presenting information in word learning studies, objects were labeled in a random order. In the other control condition (Yoked control), infants experienced a sequence of labeling events that was actively generated by another infant. This condition served to test whether learning sequences that are generated by one infant might have a degree of “objective optimality” (perhaps determined by the perceptual relationships between stimuli) that also facilitates learning in other infants compared with random presentation.

We tested 20–23‐month‐old infants, an age for which, to the best of our knowledge, no previous studies of active word learning exist. The study, therefore, provides novel insight into the mechanisms underpinning language development at an age that sees the emergence of multi‐word utterances and substantial increases in vocabulary size (McMurray 2007; Reznick and Goldfield 1992).

### 1.3 Hypotheses

We expected to observe advantages for curiosity‐driven learning on subsequent label recognition. We therefore hypothesized that infants in the Curiosity condition would show better learning of novel object labels than infants in the Random and Yoked control conditions. Such a result would provide evidence that self‐motivated information selection and the freedom to choose what to learn about (albeit without their metacognitive awareness) are important aspects of infants’ learning in the real world, optimizing their learning by reducing referential uncertainty. We further expected that infants in the Yoked condition would show slightly higher rates of learning than infants in the Random condition, based on the reasoning that such sequences are in part systematically and optimally determined (although not through an active choice of the learner), for example, by the perceptual features of the objects or their spatial location. Alternatively, if infants showed better recognition following passive learning (i.e., in the Yoked and Random conditions), or similar rates of learning across all three conditions, this would suggest that curiosity may not provide a unique advantage in infant word learning.

## 2 Method

The anonymized study data, code, and laboratory log are openly available here: https://osf.io/ndthz/. The Stage 1 IPA Registered Report protocol is deposited here: https://osf.io/dh2cy.

### 2.1 Participants

Participants were recruited from a database of families in the Northwest of England who have voluntarily expressed interest in participating in infant studies. Participating infants received a book and their caregivers were reimbursed for travel expenses, in accordance with standard laboratory practices. The study received university ethics committee approval. Caregivers were asked to give informed written consent and were free to withdraw from participation at any point. Data collection took place between April 2023 and May 2024.

Participants were seventy‐five 20–23‐month‐old infants (*M*_age_ = 21.50 months, *SD*_age_ = 1.83, 30 girls), English‐speaking, predominantly White and middle‐class, who were full‐term (i.e., born after at least 37 weeks of gestation) and who had no known developmental delay, or auditory or visual impairment. G*Power 3.1.9.7 software (Faul et al. 2007) was used to determine sample sizes to achieve 90% power and an effect size of 0.5 with a standard alpha (0.05). We chose these key parameters to ensure the study is highly powered, opting for a medium effect size due to the lack of directly relevant previous research paradigms or meta‐analyses which could justify a different expected effect size, and defaulting to the alpha level conventionally used; however, supplementing our frequentist analyses with Bayes Factors to minimize overreliance on the alpha threshold (Lakens et al. 2018).

Our calculations indicated that a sample of 54 is sufficient for the main planned one‐way three‐factor ANOVA analysis, and a sample size of 51 is sufficient for the mixed‐effects model with a binary dependent variable, based on an *Χ* ^2^‐test with df = 2. The final sampling decisions were based on the principles of Bayesian sequential testing (Mani et al. 2021; Schönbrodt et al. 2017; Schönbrodt and Wagenmakers 2018). We determined a minimum *a priori* group size of 18 infants (based on the power analyses for ANOVA) and a maximum group size of 25 infants (based on the funding constraints) for each condition. In line with the Bayesian sequential testing protocol, if after reaching the pre‐specified minimum number of participants a pre‐determined Bayes Factor (BF) threshold was not achieved, then the Bayes Factors were calculated after every new participant until either reaching the pre‐determined threshold in either direction, or testing the maximum pre‐specified number of participants. We set the Bayes Factor threshold at 10 (per guidelines set forth by Mani et al. 2021; De Santis 2004; Weiss 1997), with BF_10_ > 10 (on ANOVA) interpreted as evidence for the research hypothesis (i.e., that self‐motivated information selection supports early word learning) and BF_01_ > 10 as evidence for the null hypothesis (i.e., that self‐motivated information selection has no substantial effect on early word learning). Only the participants who met the inclusion criteria as specified below were included in the final sample; thus, recruitment continued until the sample was reached (see Figure S1).

In line with the procedure described above, the final sample consisted of 75 infants: Curiosity condition (*n *= 25; *M*_age_ = 22.14 months, *SD*_age_ = 1.00, 9 girls); Yoked (*n* = 25; *M*_age_ = 21.30 months, *SD*_age_ = 2.29, 11 girls); Random (*n* = 25; *M*_age_ = 21.07 months, *SD*_age_ = 1.86, 10 girls). Participants were excluded based on the pre‐determined criteria (See Exclusions section below).

### 2.2 Experimental Procedure

Upon welcoming the participants into the laboratory play area, the experimenter explained the study procedure to the caregivers and obtained written consent before playing with the child to familiarize them with the laboratory environment. Participants were then invited into a dedicated testing room. Testing took place in a darkened 1.8 m by 2.5 m area partitioned by black curtains, with overhead lights dimmed. The researcher remained on the other side of the curtain in the adjacent lab space.

Infants were seated in a stationary high chair or on the caregiver's lap if they did not tolerate the chair (*n *= 27) approximately 0.6 m away from a screen (Dell 2211H, with 21'' 1920*1080 LCD monitor). Although caregivers were asked not to talk or interact with their child during the experiment, and to keep their eyes closed throughout the procedure; it was made clear that they may stop the experiment if necessary at any time. A stationary eye‐tracker (Tobii Pro Spectrum) was used to capture infant looking to the screen (with a sampling rate of 1200 Hz). Sounds were played through external speakers. The entire procedure was audio and video recorded for experimenter monitoring of the procedure and offline coding of infant attention, if necessary. The overall duration of the eye‐tracking experiment was about 7 min.

Infants were assigned to one of the three conditions defined above: Curiosity, Random, or Yoked. Following calibration, the experimental procedure consisted of three phases: warm‐up, learning, and test. Warm‐up and test were identical in each condition, while the learning phase differed. In the experimental Curiosity condition, object labels were provided in a gaze‐contingent manner, while in the Yoked and Random control conditions infants had no influence over which objects were labeled.

#### 2.2.1 Gaze‐Contingent Eye‐Tracking

A novel gaze‐contingent paradigm was used to label the object on which the infant's gaze fixated (using custom‐designed software programmed in Matlab 2021, Psychtoolbox 3, and delivered via Tobii Pro SDK 1.6.1.21). Prior work has shown that even 6‐ to 8‐month‐old infants can learn to exert control in gaze‐contingent paradigms after as few as three trials (Wang et al. 2012). Throughout the procedure, an audio‐visual attention getter was displayed until infants fixated for 150 ms, which triggered the stimulus presentation. Gaze fixation on an object for at least 700 ms triggered the labeling events during the learning phase in the Curiosity condition. Gaze fixation thresholds were determined by pilot testing (*n* = 5) and are in line with published literature of similarly designed gaze‐contingent studies with infants (Sučević et al. 2021: 10‐month‐olds with 500 ms; Wang et al. 2012: 6–8‐month‐olds with 600 ms; Zettersten 2020: 20‐month‐olds with 700 ms).

#### 2.2.2 Calibration

Upon positioning an infant in front of the screen, a 20‐s video clip from a Peppa Pig cartoon was played on the screen to attract the infant's attention. Immediately following the clip, an image of Peppa Pig was used as a custom eye‐tracker calibration point, accompanied by audio prompts (e.g., “Look at Peppa!”; “Where is Peppa?”; “Where did she go?”). A five‐point calibration was used capturing gaze at each of the four corners and the center of the screen. Infants were required to fixate each point before the experimenter manually advanced the calibration sequence. If reliable calibration could not be achieved at the first attempt, the experimenter repeated it using a different image along with the audio prompts at each calibration point location change. If successful calibration still could not be achieved after three attempts, the experimenter offered participants a break, distracted the infant by playing with them (e.g., blowing bubbles), and re‐attempted the warm‐up and calibration phase again. If calibration was still unsuccessful, the experimenter terminated the study. No infants were excluded due to unsuccessful calibration.

#### 2.2.3 Warm‐Up

This phase introduced infants to the experimental set‐up, and trained those assigned to the Curiosity condition to navigate the objects on the screen using their gaze—a skill crucial for administering the subsequent gaze‐contingent learning phase in this condition. Infants assigned to the Yoked and Random control conditions received the same procedure, but their gaze was not tracked, and the events were triggered automatically after being displayed for 1 s.

**FIGURE 1:**
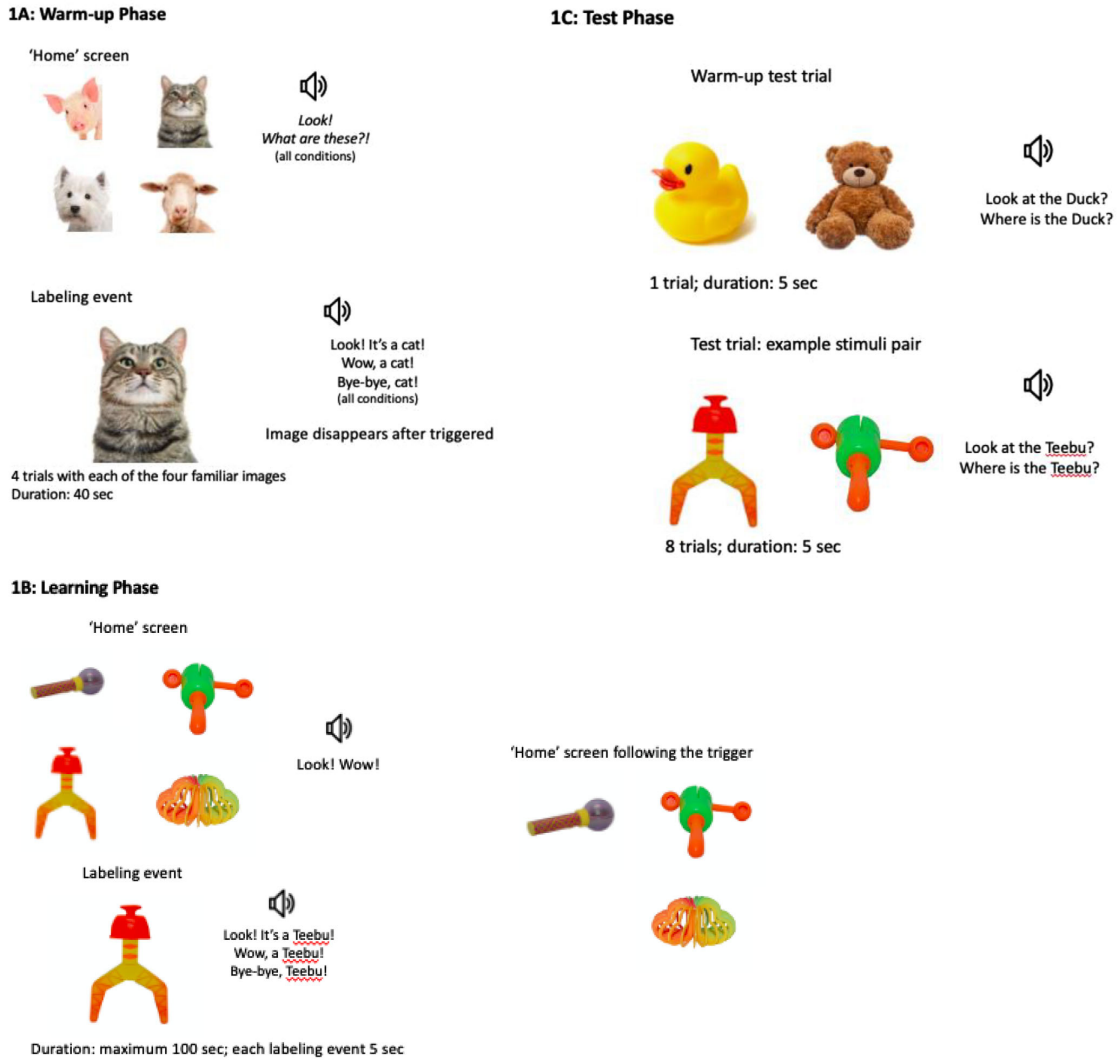
Experimental design flow and examples of stimuli.

At warm‐up (see Figure 1a), infants saw the “home” screen partitioned into four equally‐sized AOIs with one familiar object (sheep, cat, dog, cow) displayed in the center of each AOI (100*100 mm). An audio track was played to direct infants’ attention to the screen (“Look! What are these?”). Upon an infant fixating on one of the objects (in the Curiosity condition by fixating within the respective AOI for at least 700 ms, and without an infant's active choice in the control conditions), the image was enlarged to fill the screen (200*200 mm), accompanied by the verbal acknowledgement of their choice (e.g., “Look, it's a cat! Wow, a cat! Bye‐bye, cat.”). Familiarity with these labels in 20‐month‐olds was expected based on the CDI norms data (Frank et al. 2016), and was also pre‐tested with the caregiver checklist, thus serving as a pre‐screening criterion. The “home” screen with the same images except for the one triggered in the preceding trial would then return and infants were prompted to make another independent choice by attending to any of the remaining images. If children failed to trigger the last remaining image for 10 s, it was activated automatically to complete this phase.

#### 2.2.4 Learning

Immediately following the warm‐up, infants saw the “home” screen with four AOIs, identical to the four‐object display in the Warm‐up phase, but with four novel objects instead (see Figure 1b). Soft background music was played while the home screen was presented along with an auditory attention‐getter (“Look! Wow!”). A labeling event proceeded as follows: The selected object made a sound and was then enlarged. An audio track was then played, providing a label for the object three times in varying utterances (e.g., “Look, it's a Teebu!”; “Wow, a Teebu!”; “Bye‐bye, Teebu!”) for a total duration of 5 s per learning trial. The labeling event then ended with the return of the original screen with the same four novel objects. Each object received a different novel label, with identity randomized across participants (fiffin, pizer, teebu, virdex; from the Novel Object & Unusual Name (NOUN) Database 2nd Edition; Horst and Hout 2016).

In the Curiosity condition, labeling events were triggered by the infant fixating on one of the four objects for at least 700 ms (used as an index of their interest and curiosity in the absence of any cues or verbal prompts), identical to the warm‐up phase of the gaze‐contingent paradigm. This condition therefore gave infants complete control over which object they wanted labeled at a certain time and how many labeling trials occurred, and it allowed them to return to the same object should they choose to do so. However, to ensure infants had an opportunity to learn the labels for all four objects, triggering the same object twice consecutively was impossible: after triggering any object, for the duration of the next trial, that object was not displayed on the home screen, thus directing the infant's attention to one of the other three objects instead. After the subsequent trial, the previously triggered object was again visible on the menu screen so that infants could trigger it again^1^.

Infants were able to engage with the objects on the “home” screen for a maximum of 100 s to ensure they had sufficient time to select all four objects, while not guiding them to do so in any systematic manner except for restricting reengagement with the immediately preceding stimulus. The learning phase would be paused if attention could no longer be attracted to the screen, as indexed by the infant looking away from the screen for five continuous seconds. In this case, the researcher attempted to re‐engage the participant with the help of the caregiver, if necessary, for a maximum pause of 1 min. If the infant refused to continue, the study ended.

In the Random condition, infants passively experienced labeling events in a random sequence, using the mean number of labeling events triggered by infants in the Curiosity condition who sampled at least three target objects. In the Yoked condition, infants passively experienced labeling events in a sequence generated by a randomly selected infant from the Curiosity condition who triggered at least three target labeling events. We expected the majority of infants to trigger all four targets given the high saliency of images, the extensive warm‐up procedure, and the learning phase design. In this sample, the mean number of triggered labelling events was 3.5. Infants who did not trigger at least three targets were excluded from data analyses, per the exclusion criteria below. In order to generate such sequences, the Curiosity condition was run before recruiting infants into the Random and Yoked conditions.

#### 2.2.5 Test

The test phase was identical for all infants in all conditions and consisted of a total of eight trials, preceded by a single warm‐up test trial displaying two new familiar objects (duck and bear toys) to ease infants into the test procedure (see Figure 1c). A colorful central attractor image along with a sound was displayed until infants focused on it for at least 150 ms, which triggered the presentation of each pair of the test objects. In each trial the infant was presented with two of the four objects, displayed side‐by‐side forming two equally‐sized AOIs (100*100 mm). An audio track was played to ask infants for the target object using two varying utterances (i.e., “Look at the Teebu!”; “Where is the Teebu?”). Each image pair remained on the screen for 5 s, with the onset of the target label at 2.5 s, thus dividing the trial into two equal length periods: pre‐labeling (baseline preference for objects) and post‐labeling (label‐recognition‐based preference for objects). Each target image was presented twice, with order pseudo‐randomized, once on the left and once on the right of the competitor object.

### 2.3 Measures

Infants’ looking time at the presented AOIs during each phase was measured using the eye‐tracker.

#### 2.3.1 Learning

As a measure of general attention, we recorded infants’ cumulative looking times at objects during the labeling events. For infants assigned to the Curiosity condition we performed exploratory analyses of their patterns of active looking at each of the four novel objects before triggering the labeling event (i.e., the breadth of exploration), and the number of triggered labeling events.

#### 2.3.2 Test

Two complementary measures were derived from the test phase to examine infant word learning from different perspectives and to avoid over‐reliance on a single measure choice (LoBue et al. 2020). (1) The proportion of infant looking at the target image was baseline‐corrected by subtracting the pre‐labeling target proportion looking (0–2500 ms) from the post‐labeling target proportion looking (2740–5000 ms), and calculated as the post‐labeling length of looking at the target AOI divided by total looking at both objects (based on previous research, we measured post‐labeling from 240 ms post‐label‐onset to account for the timing of planning and executing saccades; Swingley et al. 1998). If infants cumulatively looked to the target above chance on both trials for the same target label, it indicated that they had successfully learned that label. (2) A trial‐level accuracy score was coded as a binary outcome: 0 – did not learn label, 1 – learned label. In both measures, we analyzed test trials only for those objects that were triggered at least once in the learning phase, so that if the child did not trigger all four images, the test trials for the non‐triggered labels were discarded from the analyses.

### 2.4 Exclusion Criteria and Data Cleaning

Only infants whose caregivers reported their knowledge of the familiar labels used in the warm‐up phase were included in the final dataset. A total of 96 infants were tested in the study. Data for 11 participants was fully excluded for the following reasons: Recording less than 30% of usable eye gaze data overall due to infants’ general inattentiveness or technical failures (*n* = 2 – Curiosity; *n* = 4 – Random; *n* = 4 – Yoked), and caregiver interference, defined as caregivers speaking to or interacting with their infants during the learning and/or test phase on more than 30% of the trials (unique labeling events) (*n* = 1 – Yoked).

Data from the test phase were partially excluded on a trial‐by‐trial basis if infants looked to the screen for less than 30% during the stimulus presentation (25 trials—Curiosity; 13 trials—Random; 12 trials—Yoked) or failed to fixate on both pictures (the target and the distractor) during the trial (5 trials—Curiosity). Infants with at least one valid test trial were included into the dataset. Overall, there were 145 valid trials in Curiosity (*M* = 5.80), 145 in Random (*M* = 5.80), and 161 in Yoked conditions (*M* = 6.44). To improve the data quality due to data loss and noise (e.g., eye blinks), data were interpolated for a maximum of 150 ms of the missing data using a simple linear interpolation. For 13 participants, due to a technical error, the sampling rate was 600 Hz instead of 1200, and we transformed the data to 1200 Hz to match the rest of the sample by interpolating a gap of one sample.

Specific to the Curiosity condition, only infants who successfully passed the warm‐up phase, defined as successfully fixating on at least three of the four images, proceeded to the learning phase. Data were also fully excluded if the infant did not trigger at least three possible labeling events at the learning phase (*n* = 10). Data collection continued until the pre‐specified, post‐exclusion examination sample was obtained.

### 2.5 Analysis Plan

Analysis consisted of two independent but complementary approaches: one‐way ANOVA and mixed effects regression modeling (Table 1). These distinct but convergent approaches were both aimed at answering the research question (does self‐motivated information selection facilitate better infant word learning?) and were motivated by the use of two outcome measures we chose to infer successful word learning at the test phase: the proportion of infant looking at the target image and the binary trial‐level accuracy score. Table 1 provides possible outcomes and interpretations for both chosen types of analyses. If we would not find convergence between these, our final interpretation would have to account for the contradictory evidence obtained using both complementary approaches, rather than weighting a single choice more heavily (LoBue et al. 2020).

**TABLE 1:** Summary of study design.

Statistical analysis	Possible outcomes	Interpretation
One‐way ANOVA with three factors (conditions) on the proportion of target looking at test, with the post‐hoc pairwise Tukey test and a corresponding Bayes Factor analysis (default prior with a wide Cauchy distribution; scale of effect = 0.707)	Target looking occurs significantly more in Curiosity condition than control conditions at *p* < 0.05 and BF_10_ > 10 Target looking occurs significantly more in Yoked and/or Random conditions than Curiosity condition at *p* < 0.05 and BF_10_ > 10 Target looking occurs at equal high rates across all three conditions at *p* < 0.05 and BF_10_ > 10 Target looking occurs at equal low rates across all three conditions at *p* > 0.05 and BF_01_ > 10	Evidence for the research hypothesis: self‐motivated information selection supports word learning. Evidence for the null hypothesis: self‐motivated information selection has no substantial effect on early word learning, while passive learning does. Evidence for the null hypothesis: self‐motivated information selection has no substantial effect on early word learning above and beyond passive learning. Evidence that the paradigm does not support word learning.
Mixed‐effects modelling: stepwise binominal logistic regression	Fixed effects and random slopes for Curiosity condition are the only significant predictors of the test trial‐level accuracy scores at *p* < 0.05 Fixed effects and random slopes for Yoked and/or Random conditions are significant predictors of the test trial‐level accuracy scores at *p* < 0.05 No fixed effect of condition is detected as a significant predictor of the test trial‐level accuracy scores at *p* > 0.05 Fixed effects and random slopes for Curiosity condition are significant predictors of the test trial‐level accuracy scores at *p* < 0.05 and BF_10_ > 10, but after controlling for other IV variables or interaction variables	Evidence for the research hypothesis: self‐motivated information selection supports word learning. Evidence for the null hypothesis: self‐motivated information selection has no substantial effect on early word learning. Evidence for the null hypothesis: self‐motivated information selection has no substantial effect on early word learning. Supporting evidence for the research hypothesis: self‐motivated information selection supports word learning when also accounting for other factors, such as, for example, the number of the triggered labeling events or inter‐individual variability.

We used a combination of frequentist and complementary Bayesian statistical approaches to provide a better characterization of our results (Dienes and McLatchie 2018). For all frequentist analyses, we used a significance threshold of *p* < 0.05. To provide evidence for either the null (which the frequentist approach is unable to do) or the alternative hypothesis, corresponding Bayesian analyses were carried out for all frequentist analyses, using a default prior with a wide Cauchy distribution (scale of effect = 0.707) calculated using the *BayesFactor* R package (Morey and Rouder 2015), and adopting the threshold of BF > 10 as evidence for substantial support for either the research (BF_10_) or the null (BF_01_) hypothesis (Jeffreys 1961).

First, we compared target looking across conditions by performing a one‐way three‐factor (Condition: Curiosity, Random, Yoked) ANOVA with the proportion of baseline‐corrected looking at the target image at test as a dependent variable. We planned to log‐transform the variable if necessary and establish if it meets the ANOVA assumptions at the condition level. If either the normality (assessed with Shapiro–Wilk test) or the homogeneity of variances (assessed with Levene's Test for Homogeneity of Variances) assumption had been violated, the nonparametric Kruskal–Wallis *H* Test would have been run instead. The post‐hoc Tukey test was be used to disentangle any found effects between the three conditions. The sequential Bayesian analysis determining our final sample size, as specified in the *Participants* section, was based on this test.

Second, we used mixed effects modelling, because it does not require the aggregation of observations and takes into account the nested structure of the data, such as participant‐level and item‐level clustering in the data. Stepwise binominal logistic regression models were fitted using the maximum likelihood function of the *glmer* function of the *lme4* package (Bates, Kliegl, et al. 2015) and compared using the likelihood ratio test of the function *anova* in R (version 4.2.1; R Core Team 2022). Necessary data transformations were performed as needed. The models were built incrementally, and include random intercepts for participants and novel object items, random slopes for condition by item, fixed effects for condition, number of labeling events, and their interaction, retaining only the statistically significant fixed effects from the respective parsimonious models. We formulated the final *a priori* model best fitting our hypotheses as (in example *lme4* syntax): Test accuracy score ∼ condition + (1 + condition| item).

Finally, a pre‐planned, exploratory time‐series analysis assessed the dynamics of the looking time data. We conducted a growth curve analysis (Mirman 2016) using the *eyetrackingR* package (Dink and Ferguson 2015). Due to the nature of these analyses, our hypothesis about which time period is more likely to support the discrimination of Curiosity and control conditions remained unspecified. Looking data from the test phase was aggregated into 40 ms bins, excluding data points where one or both eyes could not be tracked reliably. Analysis focused on the time window between 240 ms post‐label‐onset and the end of the test trial (i.e., during 2740–5000 ms of the test trials), producing time course graphs for baseline‐corrected proportional target looking for each condition. To model our data as a linear, quadratic, and cubic function of time, in line with previous work (Ackermann, Hepach, et al. 2020; Tsuji et al. 2020) we included time and its second and third polynomial, and a random effect of item on the linear and quadratic time term.

### 2.6 Deviations From Stage 1 Protocol

We tested 29 infants using the approved Stage 1 protocol (https://osf.io/dh2cy) but encountered two issues that led us to adapt the protocol. These changes concerned some of the details of the experimental procedure and did not affect the study's experimental design, sample size, analyses plan, or hypotheses.

#### 2.6.1 Low Study Completion Rate

Only 60% of infants completed the study. To mitigate, we increased the number and strategic placement of audio attention getters and verbal prompts using highly engaging infant‐directed speech; reduced the duration of the novel label exposure in the learning phase from 10 to 5 s, and at test, shortened the duration of each trial from 8 to 5 s to help sustain infants’ attention; removed the requirement for caregivers wearing sunglasses and headphones (instead telling them not to interact with their child and not to look at the screen) as many infants found these a distraction; increased the target age group and range from 18 to 20–23 months as we found 18‐month‐olds to be harder to motivate to attend to the screen‐based presentation and sit in the high chair.

#### 2.6.2 Low Number of Different Novel Object Triggers

In the Stage 1 protocol we specified that infants who did not trigger all four novel objects would be excluded, but we found that on average, infants triggered only 1.72 objects with frequently triggering the same object consecutively. To mitigate, we optimized the warm‐up phase to train infants to trigger all four possible objects. We also prolonged the learning phase from 90 to 100 s, and restricted the possibility of consecutive multiple triggers of the same object by greying out a triggered object at the next trial. We further excluded only infants who triggered fewer than three of the novel objects and thus analyzed test trials only for those objects that were triggered at least once in the learning phase. These changes in turn precluded us from performing some of the originally pre‐registered exploratory analyses of the learning phase, such as exploring the sequence of labeling events.

## 3 Results

Descriptive statistics are presented in Table 2. On average, infants triggered 3.5 objects out of 4 and received 7.87 labeling events in the Learning phase of the Curiosity condition (the Yoked and Random values were computed based on the responses in the Curiosity condition) and looked on average at 2.06 (out of three possible) objects before making the trigger. At Test, there were on average 6.01 valid trials (out of eight possible). There were no outliers.

**TABLE 2:** Descriptive statistics by condition.

	Learning phase			Test phase		
Condition	Average items per condition	Breadth of exploration per trial	Average no. of triggered events	Baseline‐corrected looking proportion at the target object	Proportion of labels retained	Average number of labels retained
	*M (SD)*	*M (SD)*	*M* [range] *(SD)*	*M (SD)*	*M (SD)*	*M (SD)*
Curiosity	3.44 (0.71)	2.06 (0.84)	7.8 [7–8] (0.41)	0.085 (0.18)	0.442 (0.50)	1.52 (0.87)
Random	3.36 (0.49)	2.54 (0.73)	8.0 [8] (0)	0.080 (0.14)	0.429 (0.50)	1.44 (0.92)
Yoked	3.56 (0.51)	2.40 (0.76)	7.8 [7–8] (0.41)	0.098 (0.12)	0.371(0.49)	1.32 (0.99)

*Note*: Breadth of Exploration in Learning Phase refers to the number of gazes toward unique AOIs in each Learning Phase trial (up to a maximum of 3). The Proportion of Labels Retained was computed on the per‐participant, per‐label basis as correctly learned labels out of all valid test trials per label, so that if two test trials were collected, accuracy on both would be taken as evidence of the label retention, and if only one trial was collected, accuracy on it would be taken as evidence of label retention. Because different infants had a different number of labels that they could retain in principle, the baseline chance level cannot be meaningfully established—and thus this statistic is purely descriptive. The Average Number of Labels Retained was computed as the number of accurate test trials out of the Average Items per Condition.

### 3.1 ANOVA

**FIGURE 2:**
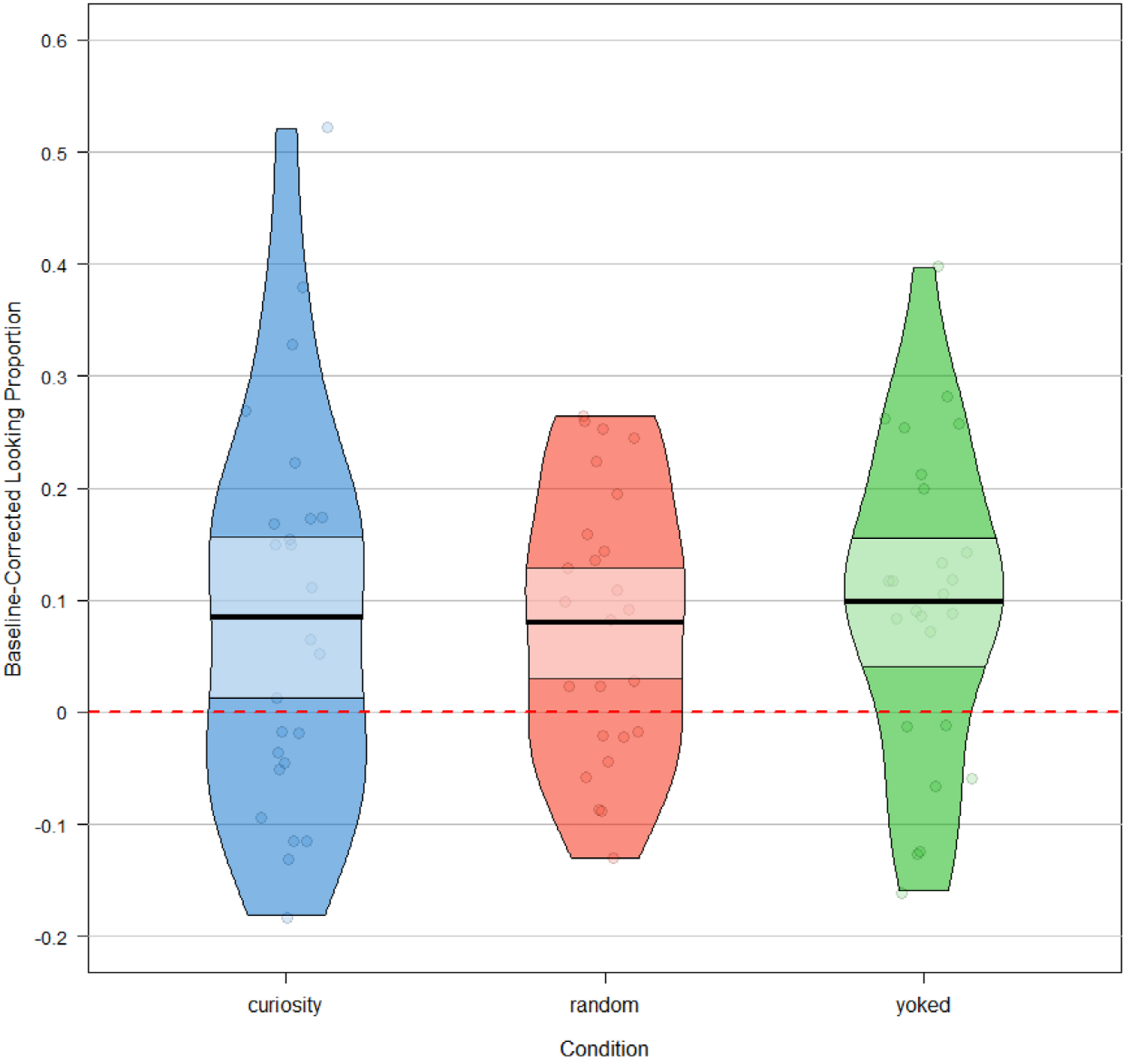
Violin plot of baseline‐corrected looking proportions by condition. Every dot corresponds to each participant's mean baseline‐corrected looking proportion at the target stimuli. The dotted red line represents the baseline‐corrected chance‐level looking proportion. Bold horizontal lines refer to mean looking proportion on that factor level; light‐colored bands represent the 95% confidence intervals.

We first compared target looking across conditions by performing a one‐way three‐factor (Condition: Curiosity, Random, Yoked) ANOVA with the proportion of baseline‐corrected looking at the target image at test as the dependent variable (see Figure 2 for a visual depiction of the results). The data met the ANOVA assumptions at the condition level and did not need to be further transformed: a Shapiro–Wilk test did not show evidence of non‐normality (*W* = 0.98, *p* = 0.246), and a Levene's Test indicated homogeneous variance between conditions, *F*(2, 72) = 1.68, *p* = 0.194. The main effect of Condition was not significant; *F*(2, 72) = 0.11, *p* = 0.900, *BF*_01_ = 8.065, with the Bayes Factor indicating moderate to strong evidence for the null hypothesis (see Figure S1 for Bayes Factor computation based on the sequential testing protocol).

### 3.2 Mixed Effects Model

A stepwise binomial logistic regression model was fitted to predict the effect of Condition on trial‐level test accuracy score at the target stimuli. We encountered a “singular fit” error even when using the minimal *a priori* theoretical model (see Table 3). This suggests that the random effect's structure specified in the model was overly complex relative to the available data (i.e., there was insufficient variance in the data to support this model; Bates, Maechler, et al. 2015). Nevertheless, the results corroborate the ANOVA analysis (and the visual inspection of the data; Figure 2), strongly suggesting the null effect of the condition (see also Supplementary Materials for the same result using the generalized linear model).

**TABLE 3:** Summary of the minimal generalized mixed‐effects model of binary test accuracy score by condition.

Fixed effects					
	Est/β	*SE*	95% CI	*z*	*p*
Intercept	0.49	0.17	[0.16, 0.83]	2.88	0.004
Random Cond.	−0.06	0.24	[−0.53, 0.41]	−0.24	0.809
Yoked Cond.	−0.08	0.24	[−0.54, 0.38]	−0.33	0.744

Random effects
	Variance	*SD*
Item (Intercept)	0.00	0.00
Random Cond. | Item	0.00	0.00
Yoked Cond. | Item	0.00	0.00

*Note*: Model equation: Test accuracy score ∼ condition + (1 + condition | item). This model was produced with a singular fit error; therefore, the results should be interpreted with caution.

### 3.3 Additional Analyses

In addition to the pre‐registered hypothesis testing analyses reported above, we performed the chance‐level analyses of performance at test. Infants demonstrated good label retention accuracy (see Table 2). One‐sample *t*‐tests on the baseline‐corrected looking time proportion outcome variable showed that label retention was above chance in all three conditions (Curiosity: *t*(24) = 2.42, *p* = 0.023, Bonferroni adjusted *p* = 0.069, *BF*_10_ = 2.368; Yoked: *t*(24) = 3.51, *p* = 0.002, Bonferroni adjusted *p* = 0.006, *BF*_10_ = 20.642; Random: *t*(24) = 3.31, *p* = 0.003, Bonferroni adjusted *p* = 0.009, *BF*_10_ = 13.610).

**FIGURE 3:**
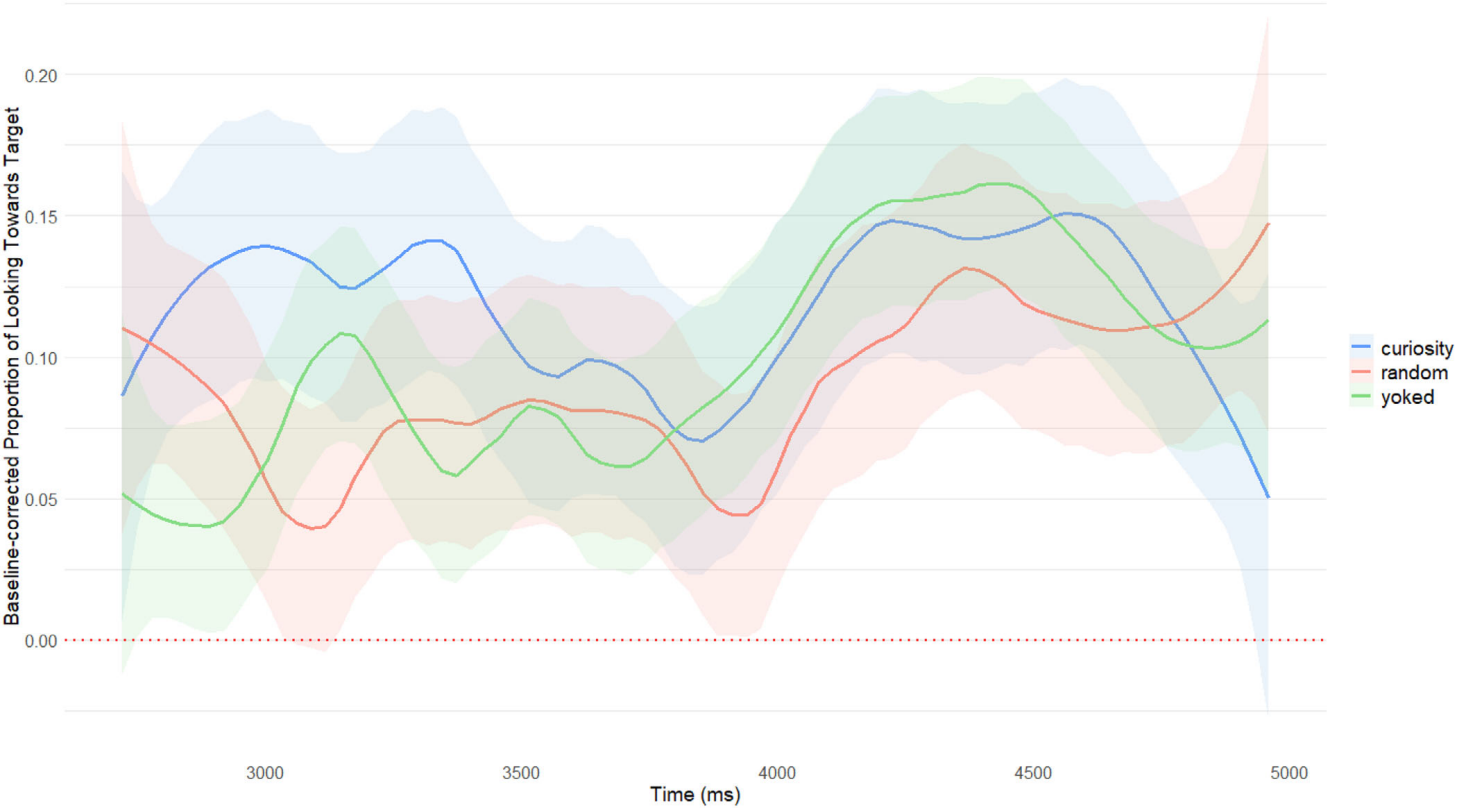
Post‐label‐onset time course of baseline‐corrected proportional target looking by condition.

The pre‐registered exploratory baseline‐corrected timeseries analysis (see Figure 3) demonstrates the lack of differentiation across conditions in the dynamic looking time at test. A growth curve analysis was fitted in accordance with the pre‐registered model, which took the form of: lmer(Elog ∼ Condition * (ot1+ot2+ot3) + (ot1+ot2|Participant) + (ot1+ot2|Item)); ot1, ot2, and ot3 correspond to the linear, quadratic, and cubic orthogonal polynomials, respectively. However, similarly to the above mixed effects regression model, we encountered a “singular fit” error, again potentially indicating a lack of sufficient variance in the shape and latency of the gaze curve between conditions.

Finally, Figures S2 and S3 demonstrate the scale of the individual differences in performance at test. It is evident that while on the group level there is no effect of condition on learning outcome, there might be individual differences not accounted for in the present study and thus worth exploring in future research.

## 4 Discussion

This study was designed to address whether curiosity‐led active information choice provides a unique advantage for infant word learning. Our results present clear evidence for the null hypothesis: self‐motivated information selection had no substantial effect on early word learning above and beyond passive learning. Further, while, as we hypothesized, participants in the Yoked condition retained novel words at a slightly higher rate than in the Random condition, this difference was not statistically significant. In fact, participants in all conditions retained novel labels above chance, suggesting that children in our study learned novel words equally well regardless of whether or not they had an opportunity to choose the referents.

The lack of difference between active and passive learning in our study contributes to the body of literature for what is a yet to be resolved question on the relationship between curiosity and learning in infancy. Curiosity and active learning have received considerable attention in developmental science, but rigorous empirical studies produced mixed results on the specific benefits of curiosity on learning outcomes. Our results do not lend support to the rather intuitive idea of the superiority of curiosity‐driven learning in infancy and early childhood (e.g., Begus et al. 2014; Kidd et al. 2012; Partridge et al. 2015; Smith et al. 2018; Lucca and Wilbourn 2018), and are not in line with indirect support provided in several recent studies, for example, that infants process information more deeply when in a state of curiosity (Chen et al. 2022). Our results also diverge from some of those obtained with older children, for example, 5–10‐year‐olds demonstrated higher retention of information following active play‐based learning as compared to learning aspects of the game in a passive condition (Stanciu et al. 2024).

Instead, our results align closely with studies showing no benefit of exploration‐based learning in children. Ackermann, Lo et al. (2020) reported that in a novel word learning task using a touchscreen‐based paradigm, 2–4‐year‐olds performed better in a passive as compared to an active learning condition. Inconclusive findings on the role of active sampling on word learning among children aged 3 years and older have also been reported by Zettersten and Saffran (2021).

Of further note, prior findings on this research question are rather nuanced. Even in the absence of a direct link between active sampling and word learning, research nevertheless demonstrated children's sensitivity to the sampling process and the context in which the novel words were acquired. In a study with 4‐year‐olds (Xu and Tenenbaum 2007), children were more likely to generalize novel labels to different categories when words were learned in a teacher‐ versus a learner‐driven (where children independently sampled objects to be labeled) condition. In a study with 4–5‐year‐olds taking place in a face‐to‐face social context (Bothe et al. 2024), while children initially demonstrated a better recall of labels corresponding to the objects they had actively sampled, this effect was transient—and with time, shifted to instead a better recognition of passively acquired labels. A study with 5‐year‐olds found no evidence of the benefits of uncertainty‐driven sampling for learning novel labels (de Eccher et al. 2024). These results suggest that while children acquire novel labels in both passive and active learning contexts, there is no clear understanding which approach leads to better learning.

Our data shows that as a group, 20–23‐month‐olds were able to learn novel labels above chance despite having limited exposure in all conditions. However, in the Curiosity condition, given the large standard deviation, we shall treat this finding with caution, due to a marginal statistical significance (if the Bonferroni correction is applied) and a complementary inconclusive Bayes Factor. Nevertheless, this is an unexpected finding, as prior research, albeit using fast mapping paradigms, showed poor label retention after a 5‐min delay despite excellent learning at referent selection phase in 24‐month‐olds with physical objects (Horst and Samuelson 2008), and with 24‐month‐olds (Bion et al. 2013) or 20–26‐month‐olds with on‐screen objects (Hilton et al. 2023). Other studies have reported the limits of learning novel labels in 16–20‐month‐olds (Mervis and Bertrand 1994), where reliable object selection was observed in only half of the sample. However, even though there are limits to infant word learning capacities (Munro et al. 2012; Taxitari et al. 2020), infants in the first year are capable of successfully learning object names from relatively few name‐object co‐occurrences (Karmazyn‐Raz and Smith 2023). The differences in the paradigms used to capture word learning might be the crucial factor in disambiguating these results.

Although we found no effect of condition and an above chance retention overall, we observed substantial variability across participants in the accuracy rate in each of the conditions. This is especially evident in the Curiosity condition, potentially leading to the inconclusive result. Emerging research on both state and trait curiosity in early infancy demonstrates that infants engage in dynamic information sampling with pronounced patterns of exploration and exploitation (Altmann, Bazhydai, & Westermann 2025) and that curiosity as a trait can be reliably detected already in infants aged 5–24 months (Altmann, Bazhydai, et al. 2025). Further, individual differences in trait curiosity appear to have an impact on later language development (Altmann 2024).

In sum, our results do not corroborate a long‐standing expectation of indexing the benefits of active learning (e.g., Gureckis and Markant 2012; Markant et al. 2016). Nevertheless, it would be premature to dismiss it and the jury is out on whether, under which conditions, and to what extent, the benefits of curiosity on learning exist. It is possible that the effects reported in the literature are specific to the age group tested, the learning domain, the method, or a combination of any of these. For example, with our design, it is impossible to confirm that infants in the Curiosity condition had indeed learned that they are in active control of the presentation of objects, although our experimental procedure was carefully designed to enable such behavior. Future research should conduct both cross‐sectional and longitudinal studies targeting different age groups, as well as test immediate versus long‐term retention. Contexts and types of information other than novel label learning should be systematically considered, such as object functions, actions, causal structures, spatial information, or information specific to the socioemotional domain, and even within the language acquisition domain, for example, verbs or more complex phrases. One interpretation of our study's null result is that word learning in this age group is particularly robust against pronounced differences in the learning contexts, which might not be the case in other learning domains. Further, presentation of novel stimuli on screen versus physical objects and measuring looking versus touching, reaching, pointing, or playing, might be manipulations worth systematically exploring in future paradigms. Finally and crucially, our study's relatively modest sample size was computed based on the expectation of a large effect size (which potentially led to a Type II error and also precluded us from running the pre‐registered analysis using mixed‐effects modelling); however, there is a possibility that the effect is smaller and a larger sample size would reveal it.

Importantly, the present study focused on self‐selected information in the absence of social interaction. It is reasonable to expect that active learning in social contexts, where novel words are acquired through infant‐initiated information seeking, such as asking for labels from social partners, or via guided play situations, might shed new light on the benefits of curiosity during dynamic communicative interactions (Begus et al. 2014; Lucca and Wilbourn 2018; Yu et al. 2018; Zettersten et al. 2024). Studies have started to address some of these questions in different age groups (Bothe et al. 2024; Eiteljoerge et al. 2024; Stanciu et al. 2024), paving the way for further investigations to capture ecologically valid active learning (Ruggeri 2022). Furthermore, the role of children's individual interests, as well as other individual characteristics, are worth considering (Ackermann, Hepach et al. 2020, Ackermann et al. 2023; Altmann 2024; Madhavan and Mani 2024; Poli et al. 2024; Rothwell et al. 2024). All these aspects present exciting new directions for advancing this line of research, with the potential to inform early childhood parenting, education, and policy.

In conclusion, our results indicate no significant differences in infants’ word learning between active and passive conditions, contrary to the hypothesis that active information selection would lead to greater novel referent retention. Instead, infants across all conditions successfully learned up to four novel object‐label associations. These results emphasize the resilience of infant word learning mechanisms and warrant further investigations of the role of curiosity in different learning contexts, opening new avenues for more fine‐grained research questions.

## Author Contributions

Marina Bazhydai: conceptualization, methodology, writing original draft, manuscript revision and editing, participant recruitment and testing, supervision, project administration, funding acquisition. Malcolm K. Y. Wong: data curation, data analyses, data visualization, participant recruitment and testing, Stage 2 methodology revision, Stage 2 manuscript revision and editing. Elena Constanze Altmann: experiment programming, participant recruitment and testing, data analyses, Stage 2 methodology revision, Stage 2 manuscript editing. Samuel David Jones: experiment programming, methodology; Stage 1 manuscript editing. Gert Westermann: conceptualization, methodology, manuscript revision and editing, supervision, funding acquisition.

## Conflicts of Interest

The authors declare no conflict of interest.

## Supporting information




**Supporting File 1**: desc70101‐sup‐0001‐SuppMat.docx

## Data Availability

All materials and the data that support the findings of this study are openly available on the Open Science Framework: https://osf.io/ndthz/. The IPA Stage 1 Registered Report Protocol is deposited here: https://osf.io/dh2cy.

## Endnotes

1 This was determined as a result of the data collection following the Stage 1 Registered Report protocol, available on https://osf.io/dh2cy, where out of 29 infants, no infant triggered all four objects, with an average of 1.72 objects triggered out of 4.
