# Supplementary material for: No Evidence for Curiosity‐Driven Information Selection Advantage in Infants’ Novel Word Learning
Source: Dev Sci. 2025 Nov 28;29(1):e70101. doi: 10.1111/desc.70101 (PMC12662157; doi:10.1111/desc.70101)
Supplement: Supplementary file 1 — Supporting File 1: desc70101‐sup‐0001‐SuppMat.docx [file DESC-29-e70101-s001.docx]

**Supplementary Materials**

**Supplementary analyses**

To confirm that the null findings revealed in the pre-registered analysis was not the result of a degenerate mixed-effects model, a simple logistic regression was computed. A generalised linear model was fitted to predict the effect of Condition (Curiosity, Random, Yoked) on the participant-level proportion of correct looks to the target image at test. The model’s goodness-of-fit was assessed using a likelihood ratio test. Compared to a null model without the predictor of Condition, there was no significant improvement in model fit, χ^2^(2) = 0.23, *p* = .890, *BF*_10_ = 0.015. This result corroborates the findings reported in the mixed-effects model, and provides converging evidence that the effect of Condition did not significantly contribute to statistical variance in the proportion of correct looks towards the target image.

**Figure S1***Bayes Factor computation with sample increase*


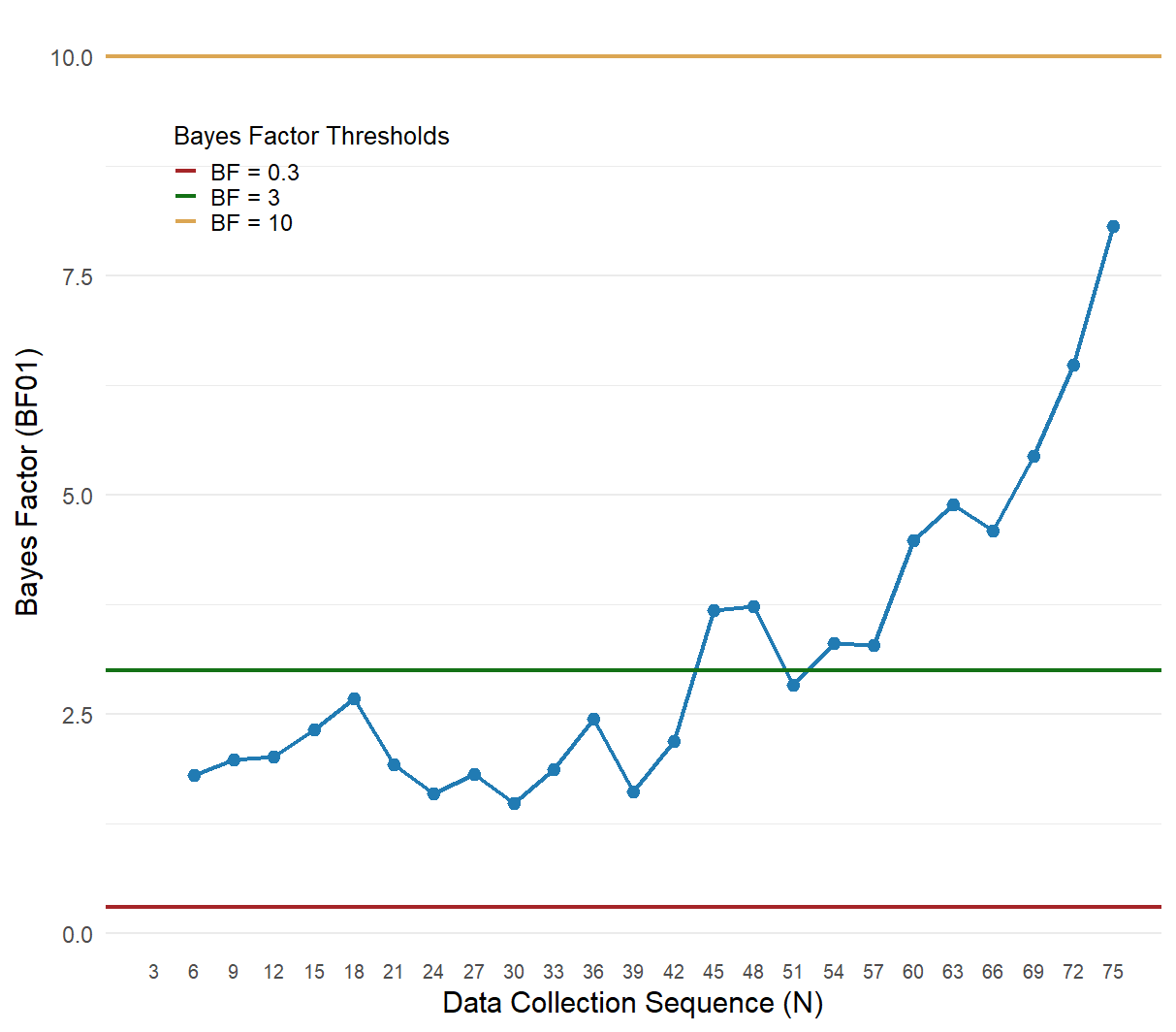


**Figure S2**


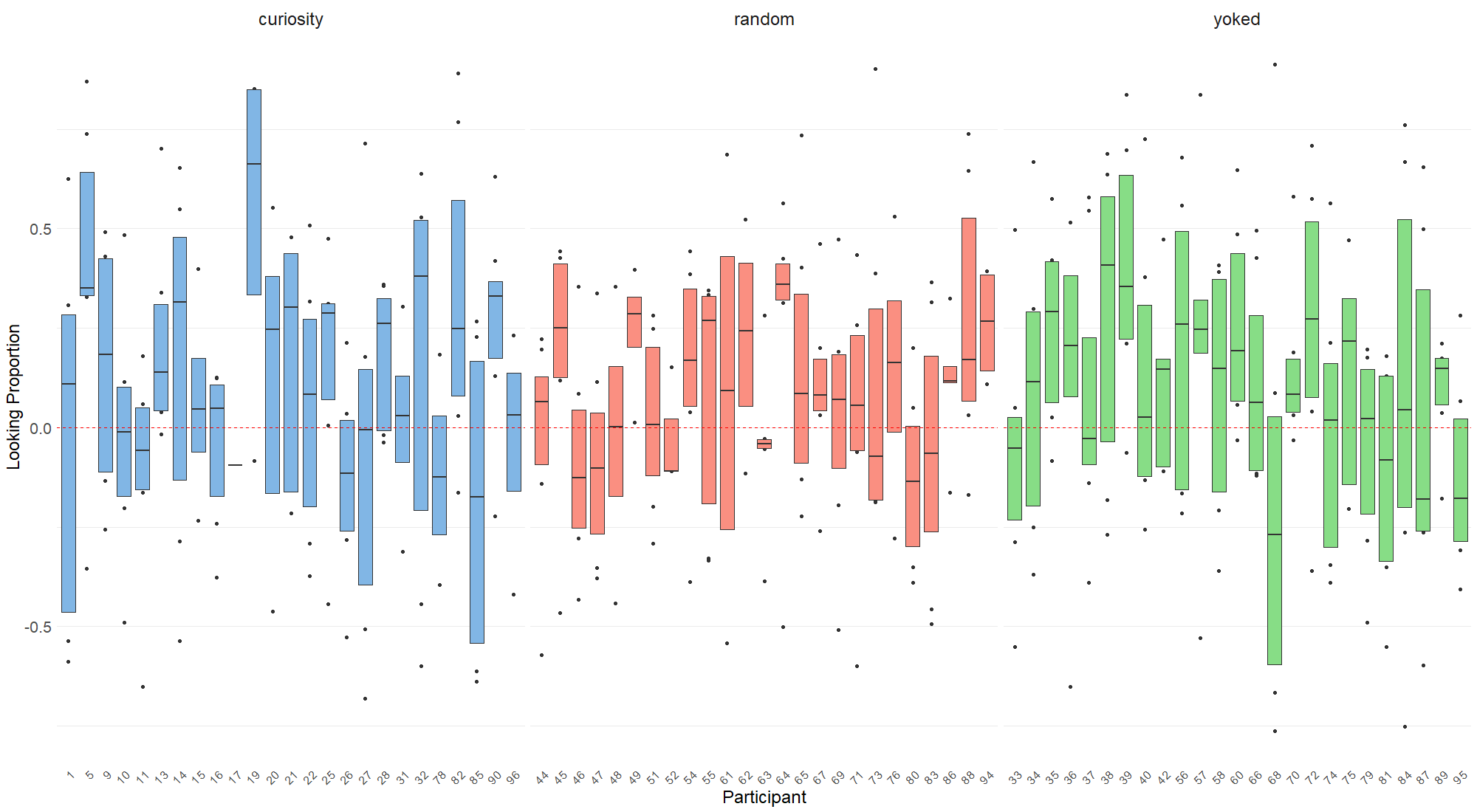
*Boxplots of Trial-level Baseline-corrected Looking Proportion at Target by Participant at Test*

*Note*. Boxes represent the interquartile range of each participant’s looking proportions, and bold horizontal lines refer to the median. Individual datapoints are represented by black dots. The dotted red line represents the baseline-corrected chance-level looking proportion.

**Figure S3**

*Boxplots of Trial-level Baseline-corrected Looking Proportion at Target by Participant at Test*


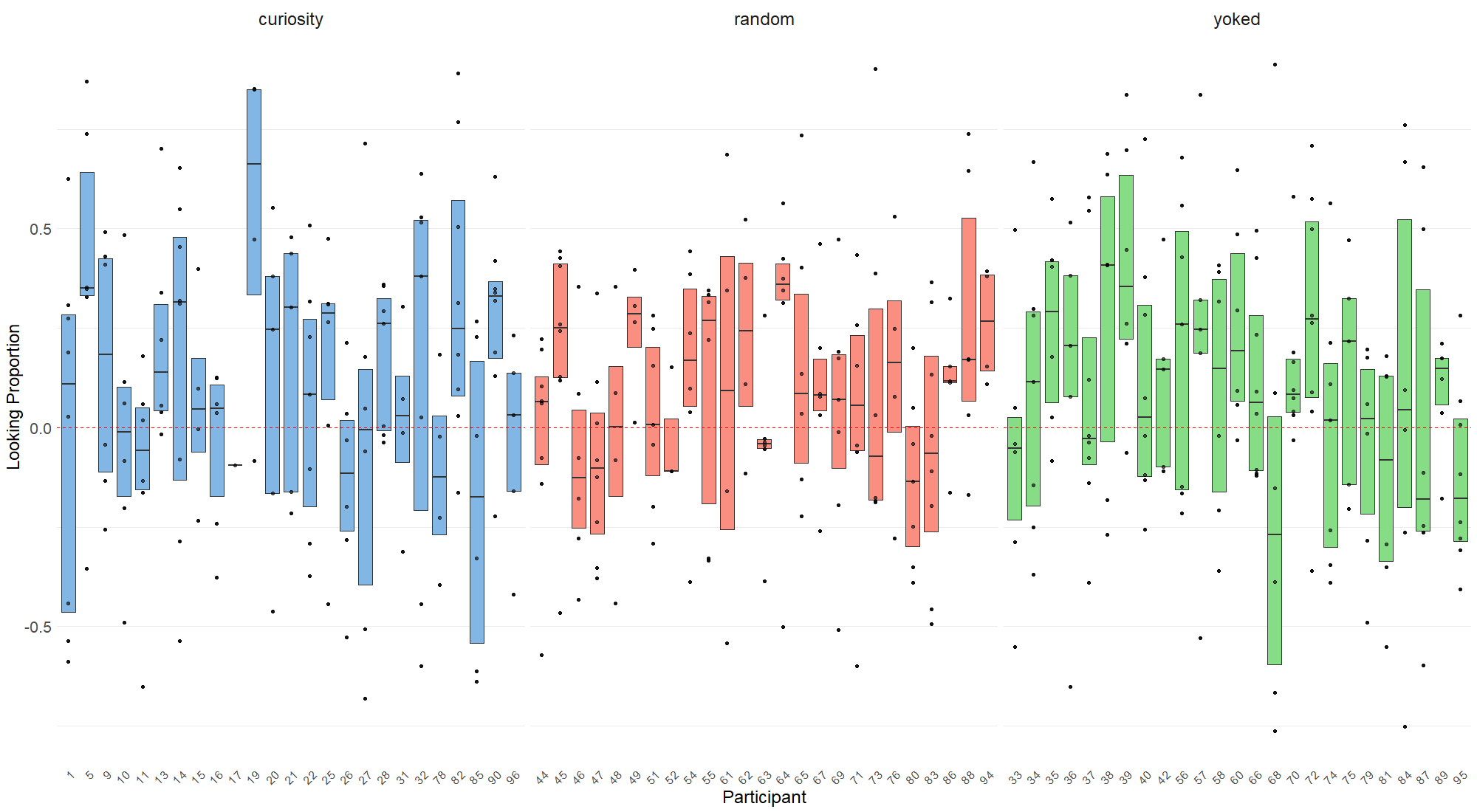


*Note*. Boxes represent the interquartile range of each participant’s looking proportions, and bold horizontal lines refer to the median. Individual datapoints are represented by black dots and include all datapoints. The dotted red line represents the baseline-corrected chance-level looking proportion.
